# Supplementary material for: Prochloraz induced alterations in the expression of mRNA in the reproductive system of male offspring mice
Source: PeerJ. 2024 Aug 26;12:e17917. doi: 10.7717/peerj.17917 (PMC11361262; doi:10.7717/peerj.17917)
Supplement: Supplemental Information 1 [file peerj-12-17917-s001.docx]

S1 The sequence data quality control statistical results of different experimental sample

| **Data**  **Groups** | **MA-1** | **MA-2** | **MA-3** | **MB-1** | **MB-2** | **MB-3** | **MD-1** | **MD-2** | **MD-3** |
| --- | --- | --- | --- | --- | --- | --- | --- | --- | --- |
| before_filtering_total_reads | 4.20E+07 | 4.66E+07 | 4.78E+07 | 4.65E+07 | 4.59E+07 | 4.51E+07 | 4.61E+07 | 4.44E+07 | 4.57E+07 |
| before_filtering_total_bases | 6.31E+09 | 6.99E+09 | 7.18E+09 | 6.98E+09 | 6.89E+09 | 6.77E+09 | 6.91E+09 | 6.65E+09 | 6.86E+09 |
| before_filtering_q20_bases | 6.18E+09 | 6.84E+09 | 7.02E+09 | 6.84E+09 | 6.73E+09 | 6.63E+09 | 6.77E+09 | 6.51E+09 | 6.72E+09 |
| before_filtering_q30_bases | 5.94E+09 | 6.55E+09 | 6.72E+09 | 6.56E+09 | 6.45E+09 | 6.37E+09 | 6.49E+09 | 6.24E+09 | 6.45E+09 |
| before_filtering_q20_rate | 9.80E-01 | 9.78E-01 | 9.78E-01 | 9.80E-01 | 9.78E-01 | 9.80E-01 | 9.79E-01 | 9.78E-01 | 9.79E-01 |
| before_filtering_q30_rate | 9.42E-01 | 9.37E-01 | 9.37E-01 | 9.40E-01 | 9.37E-01 | 9.41E-01 | 9.39E-01 | 9.38E-01 | 9.41E-01 |
| before_filtering_gc_content | 4.94E-01 | 4.92E-01 | 4.90E-01 | 5.13E-01 | 4.95E-01 | 4.98E-01 | 5.00E-01 | 4.97E-01 | 5.01E-01 |
| after_filtering_total_reads | 4.19E+07 | 4.64E+07 | 4.76E+07 | 4.63E+07 | 4.57E+07 | 4.49E+07 | 4.58E+07 | 4.41E+07 | 4.55E+07 |
| after_filtering_total_bases | 6.27E+09 | 6.95E+09 | 7.13E+09 | 6.94E+09 | 6.84E+09 | 6.72E+09 | 6.87E+09 | 6.61E+09 | 6.82E+09 |
| after_filtering_q20_bases | 6.15E+09 | 6.81E+09 | 6.99E+09 | 6.81E+09 | 6.70E+09 | 6.60E+09 | 6.74E+09 | 6.48E+09 | 6.69E+09 |
| after_filtering_q30_bases | 5.92E+09 | 6.53E+09 | 6.70E+09 | 6.53E+09 | 6.42E+09 | 6.35E+09 | 6.47E+09 | 6.21E+09 | 6.43E+09 |
| after_filtering_q20_rate | 9.82E-01 | 9.80E-01 | 9.80E-01 | 9.81E-01 | 9.80E-01 | 9.82E-01 | 9.81E-01 | 9.80E-01 | 9.81E-01 |
| after_filtering_q30_rate | 9.44E-01 | 9.39E-01 | 9.39E-01 | 9.42E-01 | 9.39E-01 | 9.44E-01 | 9.41E-01 | 9.41E-01 | 9.43E-01 |
| after_filtering_gc_content | 4.94E-01 | 4.92E-01 | 4.90E-01 | 5.13E-01 | 4.95E-01 | 4.98E-01 | 5.00E-01 | 4.97E-01 | 5.01E-01 |
| ReadsFilter% | 9.95E+01 | 9.95E+01 | 9.95E+01 | 9.96E+01 | 9.94E+01 | 9.95E+01 | 9.94E+01 | 9.94E+01 | 9.95E+01 |
| BaseFilter% | 9.94E+01 | 9.94E+01 | 9.93E+01 | 9.94E+01 | 9.93E+01 | 9.93E+01 | 9.93E+01 | 9.93E+01 | 9.93E+01 |
| low_quality_reads | 1.78E+05 | 2.21E+05 | 2.40E+05 | 1.94E+05 | 2.38E+05 | 2.32E+05 | 2.45E+05 | 2.29E+05 | 2.23E+05 |
| too_many_N_reads | 0 | 0 | 0 | 0 | 0 | 0 | 0 | 0 | 0 |
| too_short_reads | 1.26E+04 | 1.53E+04 | 1.49E+04 | 1.02E+04 | 1.71E+04 | 1.53E+04 | 1.29E+04 | 1.63E+04 | 1.42E+04 |
| too_long_reads | 0 | 0 | 0 | 0 | 0 | 0 | 0 | 0 | 0 |

Table 3 The mapping results of sequence mRNA data among different experimental sample

| **Statistics**  **Groups** | **MA-1** | **MA-2** | **MA-3** | **MB-1** | **MB-2** | **MB-3** | **MD-1** | **MD-2** | **MD-3** |
| --- | --- | --- | --- | --- | --- | --- | --- | --- | --- |
| All | 4.19E+07 | 4.64E+07 | 4.76E+07 | 4.63E+07 | 4.57E+07 | 4.49E+07 | 4.58E+07 | 4.41E+07 | 4.55E+07 |
| UnMapped | 1.61E+06 | 1.88E+06 | 2.32E+06 | 2.47E+06 | 2.32E+06 | 2.25E+06 | 2.05E+06 | 2.06E+06 | 1.86E+06 |
| Mapped | 4.02E+07 | 4.45E+07 | 4.53E+07 | 4.38E+07 | 4.33E+07 | 4.26E+07 | 4.38E+07 | 4.20E+07 | 4.36E+07 |
| MappedRate | 96.20% | 95.90% | 95.10% | 94.70% | 94.90% | 95.00% | 95.50% | 95.30% | 95.90% |
| UniqueMapped | 3.76E+07 | 4.14E+07 | 4.23E+07 | 4.12E+07 | 4.06E+07 | 4.00E+07 | 4.11E+07 | 3.95E+07 | 4.11E+07 |
| UniqueMappedRate | 89.70% | 89.20% | 88.90% | 88.90% | 88.90% | 89.20% | 89.70% | 89.50% | 90.20% |
| RepeatMapped | 2.69E+06 | 3.15E+06 | 2.97E+06 | 2.67E+06 | 2.75E+06 | 2.59E+06 | 2.68E+06 | 2.56E+06 | 2.59E+06 |
| JunctionAllMapped | 1.68E+07 | 1.86E+07 | 1.75E+07 | 1.88E+07 | 1.74E+07 | 1.66E+07 | 1.87E+07 | 1.74E+07 | 1.92E+07 |
| JunctionUniqueMapped | 1.57E+07 | 1.73E+07 | 1.63E+07 | 1.75E+07 | 1.62E+07 | 1.55E+07 | 1.74E+07 | 1.62E+07 | 1.79E+07 |
| AllBase | 5.85E+09 | 6.49E+09 | 6.65E+09 | 6.47E+09 | 6.38E+09 | 6.28E+09 | 6.41E+09 | 6.17E+09 | 6.36E+09 |
| UnMappedBase | 2.22E+08 | 2.61E+08 | 3.22E+08 | 3.43E+08 | 3.21E+08 | 3.12E+08 | 2.85E+08 | 2.85E+08 | 2.57E+08 |
| MappedBase | 5.63E+09 | 6.22E+09 | 6.33E+09 | 6.13E+09 | 6.06E+09 | 5.96E+09 | 6.13E+09 | 5.88E+09 | 6.10E+09 |
| UniqueMappedBase | 5.25E+09 | 5.79E+09 | 5.92E+09 | 5.76E+09 | 5.68E+09 | 5.60E+09 | 5.75E+09 | 5.53E+09 | 5.74E+09 |
| RepeatMappedBase | 3.74E+08 | 4.38E+08 | 4.13E+08 | 3.71E+08 | 3.83E+08 | 3.61E+08 | 3.73E+08 | 3.56E+08 | 3.61E+08 |
