# Supplementary material for: Prochloraz induced alterations in the expression of mRNA in the reproductive system of male offspring mice
Source: PeerJ. 2024 Aug 26;12:e17917. doi: 10.7717/peerj.17917 (PMC11361262; doi:10.7717/peerj.17917)
Supplement: Supplemental Information 2 [file peerj-12-17917-s002.docx]

S2 The mapping results of sequence mRNA data among different experimental sample
