# Supplementary material for: Prochloraz induced alterations in the expression of mRNA in the reproductive system of male offspring mice
Source: PeerJ. 2024 Aug 26;12:e17917. doi: 10.7717/peerj.17917 (PMC11361262; doi:10.7717/peerj.17917)
Supplement: Supplemental Information 3 [file peerj-12-17917-s003.docx]

S3 The different significantly 10 up or down gene compared expression of three different groups

| **Group MB VS MA** | | | **Group MD VS MB** | | | **Group MD vs MA** | | |
| --- | --- | --- | --- | --- | --- | --- | --- | --- |
| **Genename** | **log2FC** | **P** | **external_gene_name** | **log2FC** | **P** | **external_gene_name** | **log2FC** | **P** |
| Atp6ap1l | -2.688168276 | 1.01E-35 | Dynlt1a | -1.916787646 | 3.92E-03 | Gm7879 | -2.500081724 | 5.34E-04 |
| Rpsa-ps10 | -2.054553871 | 4.32E-29 | Olfr112 | -1.830378862 | 4.60E-14 | Dynlt1a | -2.060585715 | 2.70E-03 |
| Cwc22 | -1.990304662 | 1.05E-02 | Tpm3-rs7 | -1.728976432 | 2.10E-03 | Gm3415 | -1.713527412 | 5.15E-08 |
| Rpl30 | -1.923218736 | 1.08E-31 | Gm7879 | -1.662676152 | 2.35E-04 | Olfr112 | -1.670733055 | 3.39E-19 |
| Rsph3b | -1.603312422 | 7.09E-22 | Capza1 | -1.44369082 | 1.52E-02 | Gm3618 | -1.645882013 | 2.33E-04 |
| Ibsp | -1.29182638 | 3.66E-02 | Gm1330 | -1.276914081 | 2.44E-02 | mt-Nd6 | -1.632540494 | 3.57E-04 |
| Catsperb | -1.267997125 | 2.89E-03 | Slc26a11 | -1.253357351 | 2.91E-06 | Gm8113 | -1.508963034 | 1.72E-13 |
| mt-Nd6 | -1.266568432 | 1.03E-02 | Lcn2 | -1.225781771 | 2.65E-15 | Tpm3-rs7 | -1.46052739 | 2.02E-02 |
| Gm3409 | -1.228619312 | 1.29E-02 | Gm3448 | -1.201662841 | 5.21E-03 | Gstt1 | -1.435876749 | 7.46E-06 |
| Parp8 | -1.217066569 | 2.75E-05 | Mrps10 | -1.196786764 | 1.30E-09 | Serp2 | -1.318030334 | 4.99E-04 |
| Gm10376 | 1.703739103 | 1.33E-03 | Atp6ap1l | 2.709827039 | 4.98E-34 | Mrs2 | 3.455814192 | 1.63E-76 |
| Slc12a4 | 1.672896772 | 3.80E-02 | H2-Ab1 | 2.498057955 | 1.23E-06 | Gm14226 | 2.731445316 | 1.15E-68 |
| Pdgfrb | 1.340167678 | 5.94E-05 | Rpl30 | 2.374023346 | 2.91E-29 | Slc12a4 | 2.10288627 | 1.15E-48 |
| Lars2 | 1.301602448 | 1.63E-02 | Gm17359 | 2.085929321 | 1.20E-58 | Fam124a | 1.972226535 | 1.32E-04 |
| Tm4sf4 | 1.298661512 | 2.98E-05 | Fam124a | 1.814625218 | 8.60E-08 | Zmynd8 | 1.924382424 | 8.07E-51 |
| Psg19 | 1.197348335 | 1.24E-08 | Cwc22 | 1.781116721 | 1.54E-02 | Gm4353 | 1.840356332 | 1.04E-03 |
| Agtr1b | 1.178539508 | 6.08E-03 | Cd74 | 1.731586633 | 1.44E-02 | Gm17359 | 1.837033849 | 5.79E-67 |
| Fev | 1.175480465 | 8.04E-03 | Ubxn10 | 1.714135896 | 2.66E-04 | Hspb7 | 1.545493168 | 8.83E-14 |
| Arhgap8 | 1.118165999 | 3.29E-04 | Rsph3b | 1.656498331 | 2.86E-25 | Vrk2 | 1.431467105 | 3.26E-14 |
| Ccl27a | 1.068370736 | 1.85E-08 | Rnase10 | 1.488657908 | 9.70E-06 | Ubxn10 | 1.350905263 | 8.86E-04 |

Note： the same color indicate the same gene among groups.
